# Extensive Aeroportia, Operate or Not to Operate? A Report of a Challenging Case

**DOI:** 10.7759/cureus.13295

**Published:** 2021-02-11

**Authors:** Ejaz Latif, Khalid Ahmed, Ahmad Zarour

**Affiliations:** 1 Surgery, Hamad Medical Corporation, Doha, QAT

**Keywords:** mesenteric ischemia, acute abdomen, portal venous gas, pneumatosis intestinalis, laparotomy, splenic abscess

## Abstract

Aeroportia is the presence of gas in the portal vein. It is considered an ominous radiological sign with poor outcomes. Historically, it was associated with bowel necrosis, and surgery was mandated in all cases. Herein, we present a challenging case of portal venous gas and its management.

An 87-year-old male patient, with multiple co-morbidities, presented with abdominal pain. The computerized tomography (CT) scan showed extensive portal venous gas without evidence of bowel ischemia. Initially, he was managed conservatively, but his clinical condition deteriorated. So, an exploratory laparotomy was performed which revealed multiple superficial splenic abscesses covering the surface of the spleen and a pale segment of jejunum with questionable viability. Splenectomy was performed and second-look laparotomy was planned to assess the small bowel viability. Second-look laparotomy revealed dusky discoloration of 30 cm jejunal segment. The affected segment was resected. The patient improved after surgery and was discharged home.

In conclusion, aeroportia (portal venous gas) is a radiological entity. The clinical condition of the patient must be kept in consideration to manage the patients optimally. However, if the patient deteriorates, a high index of suspicion for mesenteric ischemia and early surgical intervention are the keys to save the patients’ life.

## Introduction

Portal venous gas (PVG) and pneumatosis intestinalis (PI) is considered an ominous radiological finding. In the past, it was associated with poor outcome. Fred et al. (1968) described a mortality of 90% in patients with PVG, however, with a better understanding of etiology and advancements in diagnostic technology and critical care, the prognosis has improved, and recently, Gonda et al. (2020) reported a mortality of 32% [1,2].

Decision making in the management of PVG/PI is still fraught with complications because there are no clear guidelines. Herein, we present a case that elaborates on this dilemma and the challenge faced in the management of the patient.

## Case presentation

An 87-year-old male of Middle Eastern descent, known to have multiple co-morbidities, presented to the emergency department with complaints of central abdominal, colicky pain which was mild to moderate in severity, non-radiating, and non-migratory. It was associated with loose stools for four days. The stool was yellow, non-bloody, and eight to nine times per day. The patient has a previous history of constipation. There was no history of vomiting, fever, or weight loss. He denies any prior such episodes.

His medical history is significant for hypertension, coronary artery disease, and asthma. His home medications included metoprolol, aspirin, valsartan, and salmeterol/fluticasone. He has no history of abdominal surgery.

Examination revealed a conscious and oriented elderly male, lying in his bed with a bit of discomfort. He had dry oral mucosa and his urine was concentrated indicating dehydration. His blood pressure was 114/70 mmHg, pulse rate was 84 per minute and regular, breathing rate was 19/ minute, temperature was 37 degree Celsius and his oxygen saturation was 96% on room air.

Abdominal examination showed no skin changes, visible scars, ecchymosis, or palpable masses. However, it was distended and mildly tender in the umbilical region without rebound tenderness. Hernial orifices were normal and bowel sounds were normal. Digital rectal examination revealed mucoid stool but there was neither blood/melena nor any palpable mass.

His lab values at presentation showed hemoglobin 12.4 g/dL (12.0 to 15.0 g/dL), white blood cell (WBC) 4 x 10^3^ per microliter (4.00 to 10.00 x 10^3^), lactic acid 1.9 mmol/L (0.5 to 1.6 mmol/L), procalcitonin 3.4 ng/mL (0.0 to 0.5 ng/mL), C-reactive protein 38 mg/L (0 to 5 mg/L), pH 7.2 (7.35 to 7.45), bicarbonate 11 mmol/L (20 to 28 mmol/L), partial pressure of carbon dioxide (PCO2) 25 mmHg (35 to 45 mmHg), creatinine 193 micromoles/L (50 to 98 Micromoles/L), blood urea 11.6 mmol/L (2.5 to 6.7 mmol/L) and albumin 24 gm/L (35 to 50 gm/L). Serum pancreatic amylase and lipase were normal. His electrolytes and liver function tests were within the normal range. A bedside urine dipstick test was negative for nitrites or leukocyte esterase.

**Figure 1:**
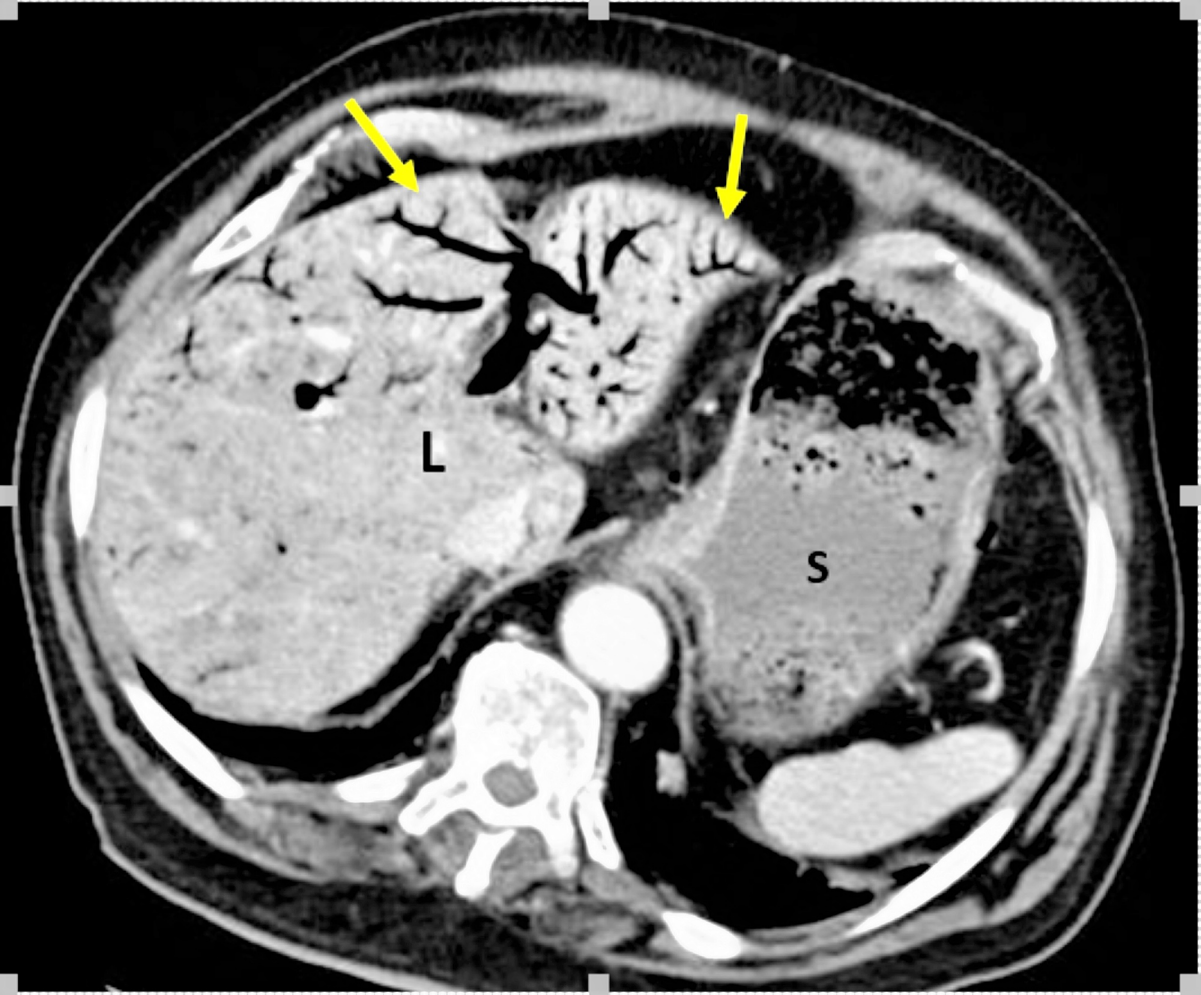
Contrast-enhanced CT scan of the Abdomen, axial view, L shows liver with yellow arrows pointing to the branching pattern of portal venous gas. S shows a dilated stomach with food contents

A chest X-ray showed no pneumoperitoneum. A CT angiogram showed gas within the tributaries of the superior mesenteric vein extending to the portal vein with extensive branching portal venous gas pattern in the liver (Figure 1).

**Figure 2:**
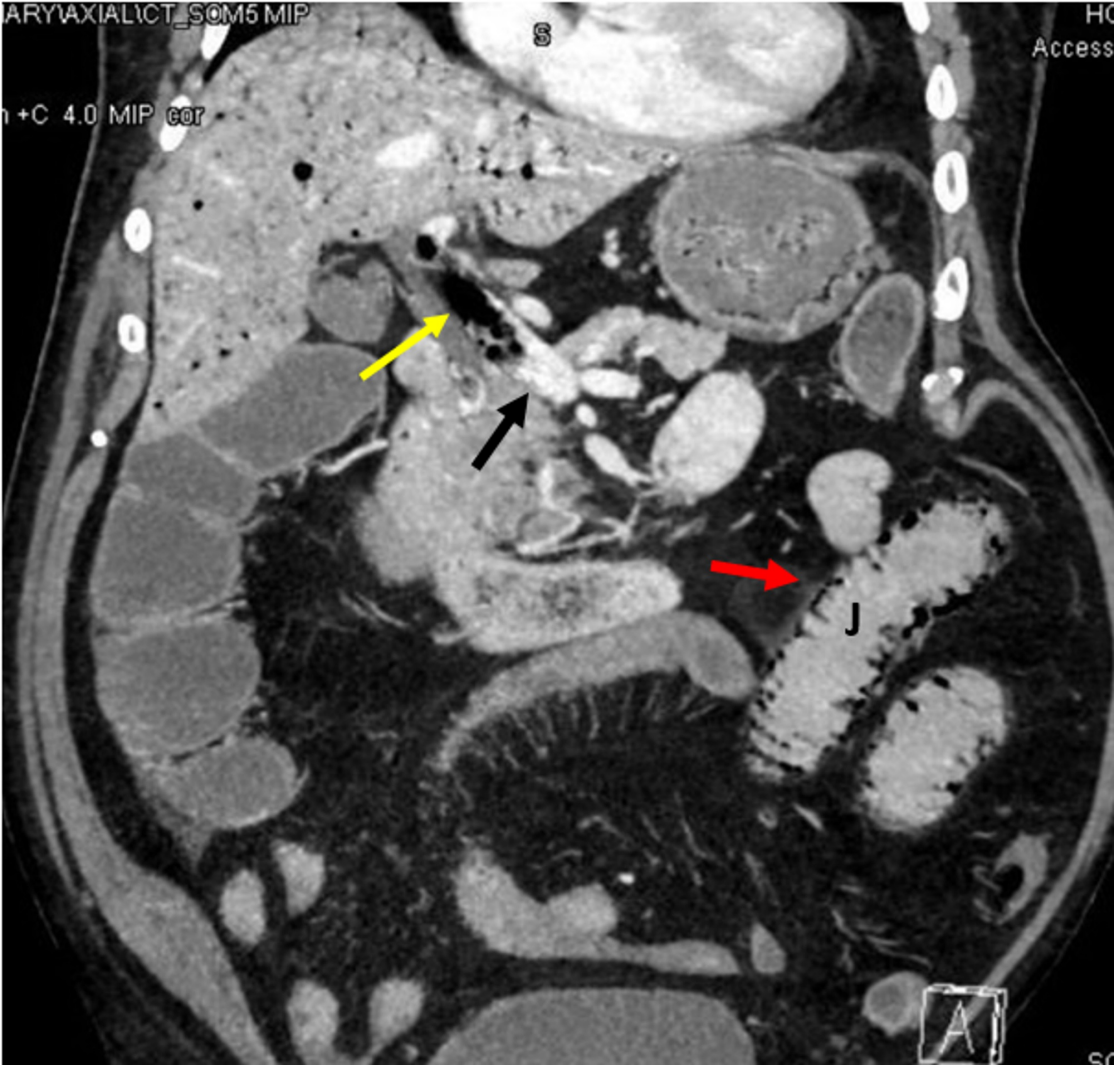
Coronal view CT scan, the black arrow denotes portal vein with gas bubbles labeled by the yellow arrow. The red arrow shows pneumatosis intestinalis in the wall of the jejunum (J).

Pneumatosis intestinalis was noted predominantly in the jejunal loops (Figure 2).

There was dilatation of the jejunum and proximal ileum without transition point. The terminal ileum and colon were collapsed. There was no pneumoperitoneum and the bowel loops were enhancing. The aorta and its major branches showed good opacification and no definite thrombus or occlusion could be demonstrated.

The patient was resuscitated with intravenous fluids. He was kept on strict input and output charting and vital signs monitoring. The patient improved clinically after fluids. Since he was not in peritonitis and clinically stable, we chose to observe him with serial examinations every two hourly by a senior surgeon. Later, after a period of 12 hours, he started to deteriorate with rising lactate levels and worsening of acidosis. He became hypotensive and was started on inotropes. However, his abdominal examination remained unchanged.

**Figure 3:**
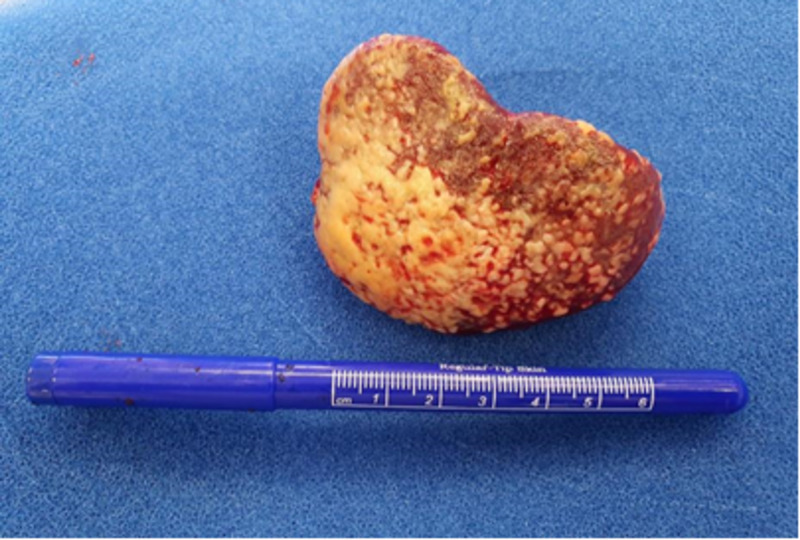
Spleen covered with thick white exudates

At this point, the decision was made to proceed with emergency exploratory laparotomy. Intraoperatively, the small bowel was dilated. The spleen was covered with white exudate giving the appearance of splenic abscess (Figure 3).

There was a 20-25 cm segment of mid-jejunum which was pale with sluggish peristalsis but viable. Splenectomy was performed and this segment of small bowel with questionable viability was kept for re-look after 24-48 hours. The patient was started on heparin infusion after the surgery.

**Figure 4:**
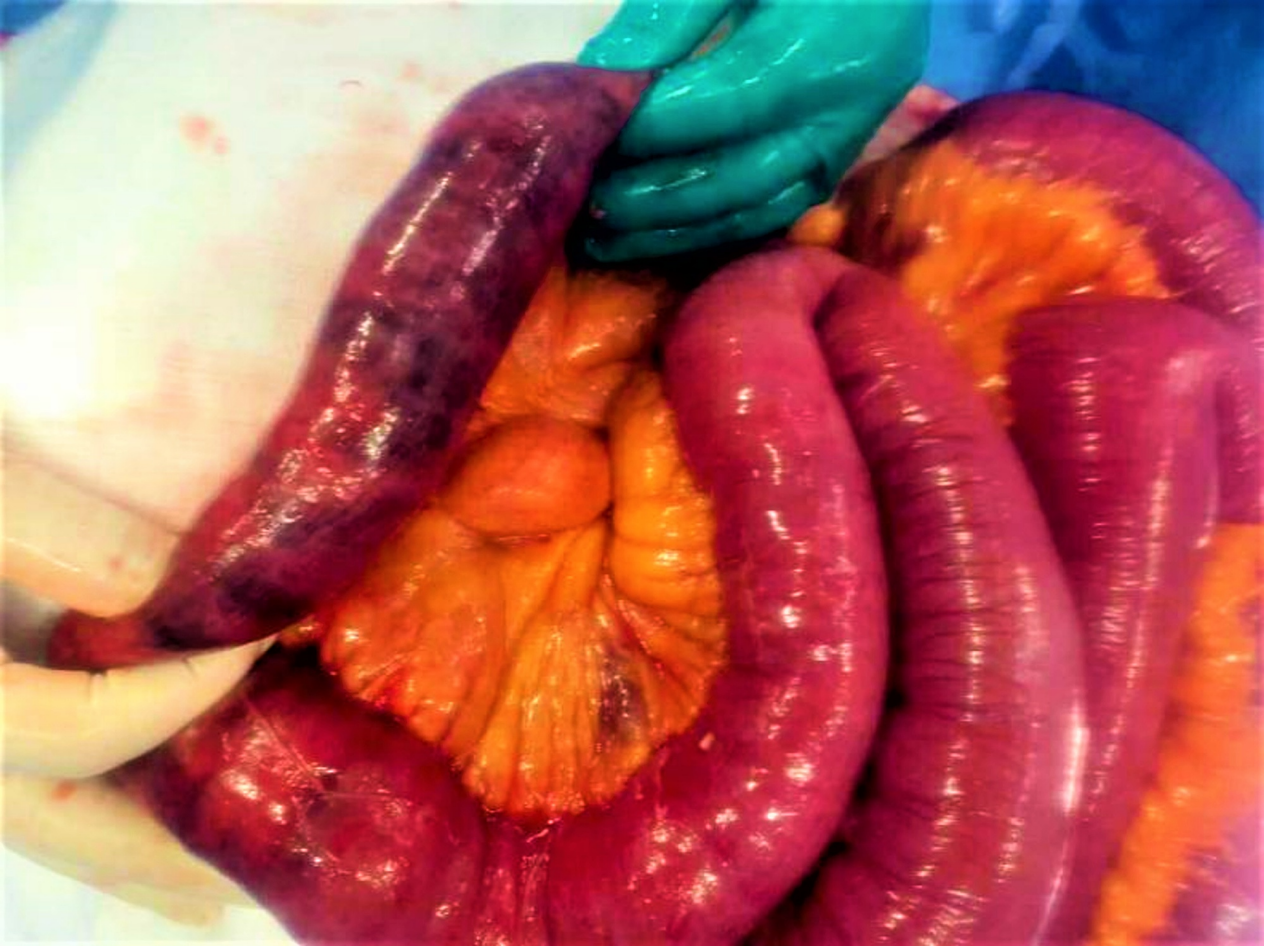
Segment of jejunum showing patchy areas of ischemia

At re-look laparotomy, this segment failed to regain its normal color and peristalsis (Figure 4). Resection of this segment was performed and end to end anastomosis was made using a gastrointestinal anastomosis (GIA) stapler.

After surgery, the patient improved hemodynamically and vasopressors were stopped on post-operative day one. The patient was extubated on day two. He was started on sips of water and eventually progressed to a full diet by postoperative day five. He was discharged after 17 days and before discharge, he was vaccinated against encapsulated organisms according to the local post-splenectomy immunization protocol. His histopathology showed the spleen with congested red pulp covered with thick white exudate. The intestinal segment showed extensive hemorrhagic infarction and necrosis of mucosa, consistent with ischemia without any vascular occlusion.

He was followed up in the clinic after two weeks and showed remarkable full recovery.

## Discussion

Acute mesenteric ischemia (AMI) is a life-threatening surgical emergency that carries significant morbidity and mortality. The usual presentation of AMI is “pain out of proportion” which may not be present in all cases [3]. One of the atypical presentations is portal venous gas, in an otherwise non-tender abdomen.

Portal venous gas (PVG) was first reported in neonatal abdominal radiographs by Wolfe et al. in 1955 [4], whereas in adults, it was reported by Susman et al. in 1960 [5]. Its true incidence is unknown. Although initially it was associated with grim prognosis because only plain radiographs were used for imaging, however, with the advancements in diagnostic radiology and as the use of multidetector CT scan became widespread, the outcome of PVG has changed. The main reasons were first, its association with non-lethal diseases became evident, secondly, it led to early surgical intervention in bowel ischemia, and thirdly, surgery was avoided in non-lethal conditions [6].

Pneumatosis intestinalis (PI), was first described by Du Vernoy in 1730. It has two subtypes, primary and secondary. Primary/idiopathic type (15%), which is often termed pneumatosis cystoides intestinalis (PCI), refers to air pockets that imply a chronic and benign idiopathic etiology. On the other hand, secondary type (85%), refers to radiological findings of linear, micro-vesicular, or more circumferential appearing intramural gas has many underlying causes, ranging from benign to life-threatening conditions like bowel ischemia. PI is a rare entity with a reported incidence of 0.03% [7]. PVG and PI share the same etiology, that is why, the terms are often used together.

The exact mechanism of PVG/PI remains poorly understood. However, certain theories may explain this phenomenon [8].

Intestinal wall permeability

A disruption of gastrointestinal mucosa allows intraluminal gas to pass into the mesenteric venous system. It is most commonly associated with bowel ischemia or obstruction.

Bowel distension

An increase in luminal gas pressure, secondary to bowel obstruction or endoscopic exams permits passage of luminal gas into the portal venous system.

Sepsis

Bacterial contamination of the portal venous system with a production of gas directly in the mesenteric system. It has been described in diverticulitis, perforated gastric ulcer, pancreatitis, and other inflammatory diseases of the bowel.

The etiology of PVG/PI can be summarized in Table 1 [9]. 

**Table 1:** Etiology of PVG/PI PVG: Portal venous gas; PI: Pneumatosis intestinalis

Inflammatory	Sepsis	Iatrogenic	Pediatric	Others
Ulcerative colitis	Abdominal tuberculosis	Post endoscopy	Necrotizing enterocolitis	Blunt trauma
Crohn's disease	Necrotizing enterocolitis	Gastrostomy	Hirschsprung's disease	Mesenteric infarction
Sigmoid diverticulitis	Suppurative cholangitis	Barium enema	Collagen vascular disease	Intestinal obstruction
Acute appendicitis	Intra-abdominal abscess	Sclerotherapy for gastric varices	Hypertrophic pyloric stenosis	Gastric ulcer disease
Acute pancreatitis		Endoscopic sphincterotomy		Paralytic ileus
		Gastric dilatation		Caustic ingestion
				Colchicine toxicity
				Seizure
				Idiopathic
				hepatic transplantation

Diagnosis

The imaging modality of choice is the CT scan, as it may also identify the underlying cause of PVG. It appears as tubular lucencies branching from the porta hepatis to the edge of the liver. These lucencies are caused by the accumulation of gas in the portal vein where the centrifugal flow of blood carries the gas to the hepatic periphery. It must be differentiated from the presence of air in the biliary tree, also known as “pneumobilia” which is characterized by the central presence of gas. Pneumobilia occurs in large bile ducts at the hilum, due to the centripetal flow of bile [6,10].

Plain x-rays like erect chest and abdominal x-rays are the usual initial diagnostic tests. It may show branching, tubular lucencies extending from the porta hepatis to the edge of the liver and identify free gas under the diaphragm. But it is not always present [1]. The M mode Ultrasound may show “meteor shower” sign which denotes flowing air bubbles in the portal vein [11].

Management

The management of PVG is controversial because it’s a radiological entity and managing such cases without considering the clinical condition can lead to a fatal outcome. Historically, surgery was advocated in all cases of PVG as it was associated with high mortality and the etiology was poorly understood. It was then questioned whether we really need to operate in all cases of PVG. This was first answered by Schulz et al., who described successful non-operative management in PVG in iatrogenic cases [10].

This shows that PVG per se doesn’t determine the decision algorithm. It is however the clinical status and underlying condition leading to PVG, which dictates the treatment. This notion is supported by Wayne et al. (2010), the largest series to date ( n=88), proposing surgical intervention for suspicious cases of mesenteric ischemia and non-operative management in benign causes [12]. However, when non-operative management is considered, same maneuvers like serial examination and frequent reassessments should be done.

In our case, the patient had mild abdominal pain and the CT scan of the abdomen although, it showed extensive PVG, the bowel was enhancing, and vessels were patent. That’s why we chose to observe him closely. But as soon as his clinical condition deteriorated, we took him immediately for surgery.

Another challenge we faced, was the intraoperative finding when we identified superficial splenic abscess and a questionable segment of ischemic bowel. The question was which one to take out first and which one is contributing to his septic condition! The bowel was pale with sluggish peristalsis, but not obviously ischemic. So, we decided to keep the questionable segment of the small bowel for re-look and to perform the splenectomy. At re-look laparotomy, the segment of bowel remained unchanged, so we decided to do bowel resection and anastomosis. One possible explanation of ischemia of the small bowel is a low flow state as our patient was in sepsis or venous micro thrombosis.

Various authors have described a management algorithm, but none seem perfect. Nelson et al. described the aggressive, be careful and conservative (ABC) algorithm, in which operative management (aggressive treatment), close monitoring (be careful), and medical treatment (conservative treatment) are based on the clinical condition of the patient. However, if PVG was detected on x-rays, he suggested for surgical intervention, which is considered obsolete [13]. Yoo S-K et al. advocated using the acute physiology and chronic health evaluation (APACHE-II) score in the management decision of PVG, as a high APACHE-II score was identified as a predictor of mortality in patients managed conservatively. But no clear algorithm was described [14]. Wayne et al. described the management algorithm of PVG taking clinical status and vascular disease score calculation into consideration [12].

**Figure 5:**
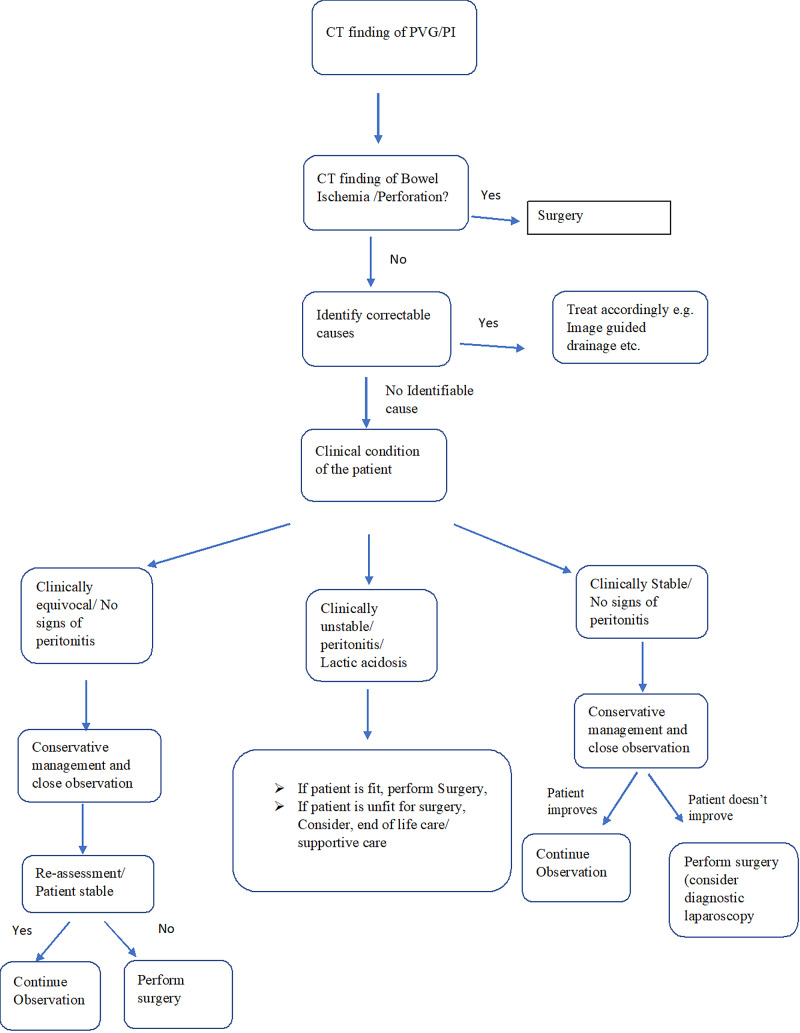
Algorithm for the management of Portal venous gas/ Pneumatosis Intestinalis

We propose a simple management algorithm that helps in the decision-making process, shown in Figure 5.

## Conclusions

PVG is a rare radiological entity, However, whenever it is found, bowel ischemia must be kept in the differential diagnosis. This case highlights the importance of clinical correlation in the management of PVG. The learning point is that a surgeon should be vigilant of this entity and in case we choose to observe our patients, frequent assessments should be performed. At any point, If the clinical condition of the patient deteriorates, the threshold for exploration should be low in order to avoid delay in management.
